# Annotations

**Published:** 1907-08-17

**Authors:** 


### Annotations.

### The Range of Clinical Observation.

One of the most impressive features of modern medical practice is the enormous increase in the adoption of objective methods in the examination of the patient. The pulse, the tongue, the temperature of the skin, and the general physiognomy, with, perhaps, a superficial examination of the urine and fæces, constituted at one time the only sources of evidence available to the physician apart from the patient's own account of his sufferings or disability. Thanks to the introduction of various instruments of precision, the objective area of examination has been greatly increased, and thus much greater exactness has been lent both to diagnosis and to treatment. Of course even the stethoscope and the clinical thermometer are comparatively recent developments, but they are now so well-established and so universally adopted that no one questions the wisdom, or even the necessity, of their use in practice. Of later date we have the laryngoscope, the ophthalmoscope, and the otoscope. Utilised at first for limited and specialised branches of practice, experience has gradually but conclusively shown that they are frequently able to throw light upon the larger and more general questions which confront the practitioner. Much the same may be said of the more modern applications of the microscope to clinical work, aided as this has been by the introduction of the various staining agents. From all this it follows that these specialised developments cannot with safety be omitted from the equipment of the practitioner. He must, in short, live up to his opportunities, and many cases nowadays cannot possibly be adequately examined or treated without investigation by the methods indicated above. The difficulties are not so great as is commonly imagined, and they may be overcome by resolution and patience.

### Anæsthetics.

Following on the publicity recently afforded in these columns and elsewhere to the question of fatalities during anæsthesia, it is interesting to note the opinions expressed at the recent meeting of the British Association for the Advancement of Science. The President of the Physiology section (Dr. Waller) devoted a large part of his address to the effects of chloroform on the human economy, and especially to the various percentage methods of administration. In view of certain opinions as to the toxicity of very slightly impure chloroform, Dr. Waller's statement is a noteworthy one, that he has tested purified chloroform against the concentrated residue of its impurities, and finds the former by far the more powerful. A difference of opinion as to the necessity for the regulation of percentage dose during chloroform anæsthesia was made evident during the discussion. The President, Professor Harcourt, and other physiologists, with Sir Victor Horsley, are convinced of it, whereas the only practising anæsthetist who contributed to the debate, Dr. Hewitt, holds the opposite view. The latter lays chief stress on two points calculated to reduce the mortality rate: one, the better education of medical students; the other, restriction of anæsthetic practice in hospitals to specialists in that field. Dr. Hewitt seems to have created some degree of sensation among the physiologists by a remark on the occasional bad effects of under-dosage with chloroform. He was, indeed, pressed for an explanation, which he gave in terms perhaps somewhat less lucid than we are accustomed to expect from him. We should have thought that the danger of shock in an extensive abdominal operation performed under very light anæsthesia, and its avoidance by pushing the administration, were sufficiently admitted now to cause no surprise in the Physiology section of the British Association.

### Poisoning by "Kaloo" Nuts.

There has recently been much interest aroused in South-East London by an epidemic of gastric symptoms amongst children and others who purchased certain nuts which were on sale on costermongers' barrows. The first cases to attract attention, five in one day on the Saturday before August Bank Holiday, were treated in Guy's Hospital; and it was largely due to the energy of the house physician, Dr. A. W. Morton Palmer, that the origin of the epidemic was discovered. Those who ate the nuts were seized very shortly afterwards with violent vomiting comparatively free from pain; the vomiting persisted for an hour or two after ejection of the partly-digested nuts, and by next day the patients seemed quite well. The vomit itself was bile-stained, but contained no blood; intestinal symptoms were few or absent. Active measures have been adopted to stop the spread of the mischief, and the whole of the nuts that can be traced have been confiscated by the authorities. Most will be, or have been, destroyed; but some have been kept for physiological and pharmacological investigation. Their active principle is not known; it may of course be that the nuts had decomposed, and that the decomposition products and not the nuts themselves produced the symptoms. "Kaloo" nuts are said not to have reached this country in bulk before. There is an oil obtained from a variety of similar nut in the East, known as "kekune oil"; possibly "kaloo" is a Borough corruption of "kekune." The nuts are the product of an Eastern tree called *Aleurites Fordii*, and there are various kinds of *Aleuritides*. The name is derived from the Greek ἄλευρον, which means meal, especially wheaten flour, and related to ἀλεῖν, to grind; from this one would expect to hear that the nuts were good for food, and might be ground up into a kind of flour. The plants belong to the natural order *Euphorbiaceæ*, and the most important species is *Aleurites triloba*, known as the "candle-berry tree"; it grows to a height of 30 to 40 feet, and is a native of the Moluccas. It is cultivated in many tropical countries for its nuts, which abound in oil, and, when dried, are used by the Polynesian Islanders as a substitute for candles hence the name "candle-nuts" or "candle-berries." The oil expressed from the kernels dries rapidly, and is known as artist's oil, or the "kekune oil." *Aleurites cordata* is the Chinese varnish-tree, and the oil from its seeds is used in China in painting.

